# Revisional Surgery for Hallux Valgus with Serial Osteotomies at Two Levels

**DOI:** 10.1100/tsw.2011.66

**Published:** 2011-03-22

**Authors:** Jason B. T. Lim, James S. Huntley

**Affiliations:** ^1^ University of Glasgow (Medical Student), Glasgow, UK; ^2^ Consultant Orthopaedic Surgeon, Orthopaedic Department, Royal Hospital for Sick Children (Yorkhill), Glasgow, UK

**Keywords:** hallux valgus, corrective osteotomy, Chevron, Scarf

## Abstract

The aetiology and form of hallux valgus (HV) is varied with many corrective procedures described. We report a 39-year-old woman, previously treated with a Chevron osteotomy, who presented with recurrent right HV, metatarsus primus varus, and associated bunion. Osteotomies were performed at two levels as a revisional procedure. This report highlights (1) limitations of the Chevron osteotomy and (2) the revisional procedure of the two level osteotomies: (i) proximal opening-wedge basal osteotomy and (ii) distal short Scarf with medial closing wedges. If a Chevron osteotomy is used inappropriately, for example, in an attempt to correct too large a deformity, it may angulate laterally causing a malunion with an increased distal metatarsal articular angle. Secondly, it is feasible to correct this combined deformity using a combination of proximal opening-wedge and distal short Scarf osteotomies.

## INTRODUCTION

Hallux valgus (HV) is a multicomponent deformity affecting the forefoot with lateral deviation of the great toe. The aetiology is varied and many surgical procedures have been described[1,2]. The principal indication for surgery is pain arising from the medial eminence of the widened forefoot. However, the subsequent rate of patient dissatisfaction may be high[3]. Deformity and difficulty with footwear are often lesser considerations and surgery for cosmetic purposes is often deferred until the onset of significant symptoms.

We report a 39-year-old woman, previously treated with a Chevron osteotomy, who presented with recurrent HV, metatarsus primus varus, and associated bunion. Osteotomies were performed at two levels as a revisional procedure. This report highlights (1) limitations of the Chevron osteotomy and (2) the revisional procedure of the two level osteotomies: (i) proximal opening-wedge basal osteotomy and (ii) distal short Scarf with medial closing wedges.

## CASE PRESENTATION: MEDICAL HISTORY, CLINICAL FEATURES

A 39-year-old woman presented to the orthopaedic clinic 5 years after a previous Chevron osteotomy. She had recurrent right HV, metatarsus primus varus, and an associated bunion with tenderness. Her right forefoot was short and wide, but there was no transfer metatarsalgia and no hypermobility of the first ray.

### Radiological Investigations

Radiology (Fig. 1) shows HV (hallux valgus angle [HVA] 37°), primus metatarsus varus (intermetatarsal angle [IMA] 22°), and a distal metatarsal articular angle (DMAA) 35°. The sesamoids are also laterally translated.

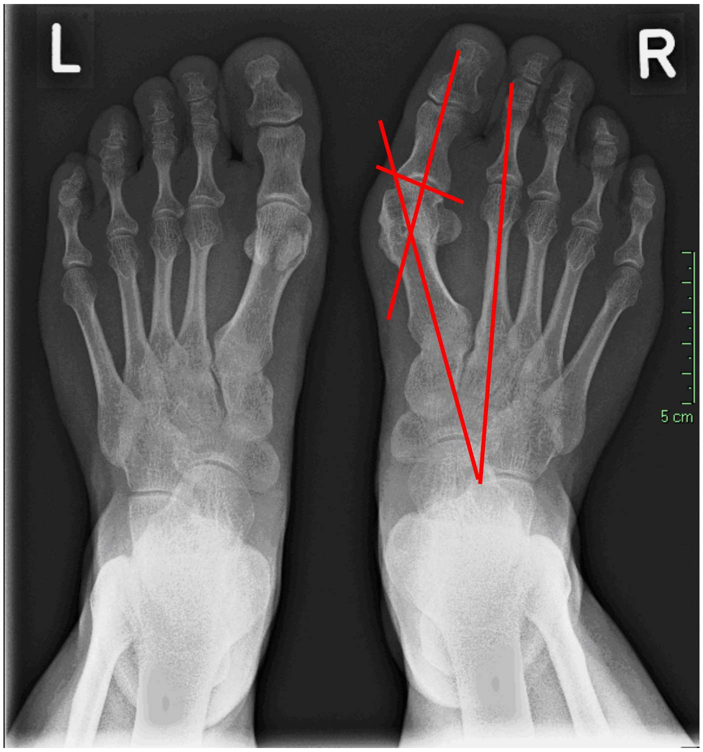

**FIGURE 1.** Weight-bearing anteroposterior views of both feet. HVA (between the long axes of the first metatarsal and the proximal phalanx) 37°; IMA (between the long axes of the first two metatarsals) 22°; DMAA (between the transverse axis defining the first metatarsal distal articular surface and perpendicular to the long axis of the first metatarsal) 35°.

### Treatment

Revisional surgery involved a lateral release — full lateral capsulotomy and detachment of adductor hallucis tendon at the base of the proximal phalanx — via a first web-space incision, followed by exostectomy, basal opening-wedge osteotomy (bone graft from exotosis, fixed with two BOLD compression screws [Integra LifeSciences Corporation, Plainsboro, NJ]). At this stage, there was intraoperative assessment by image intensifier of both IMA and DMAA (in excess of 40° lateral tilt). Therefore, a distal short Scarf procedure[4] was performed with superior and inferior 20° medial closing wedges. This was fixed with a single BOLD screw.

## Outcome and Follow-Up

After surgery, she had her foot elevated overnight. The dressings were removed at 24 h and the foot was placed in a Sabot cast (Fig. 2) to allow heel weight bearing with crutches (6 weeks). Her 3-month postoperative radiology shows correction of her HV and primus metatarsus varus (Fig. 3). There was no bony tenderness over the first metatarsophalangeal (MTP) joint and the forefoot width was substantially reduced.

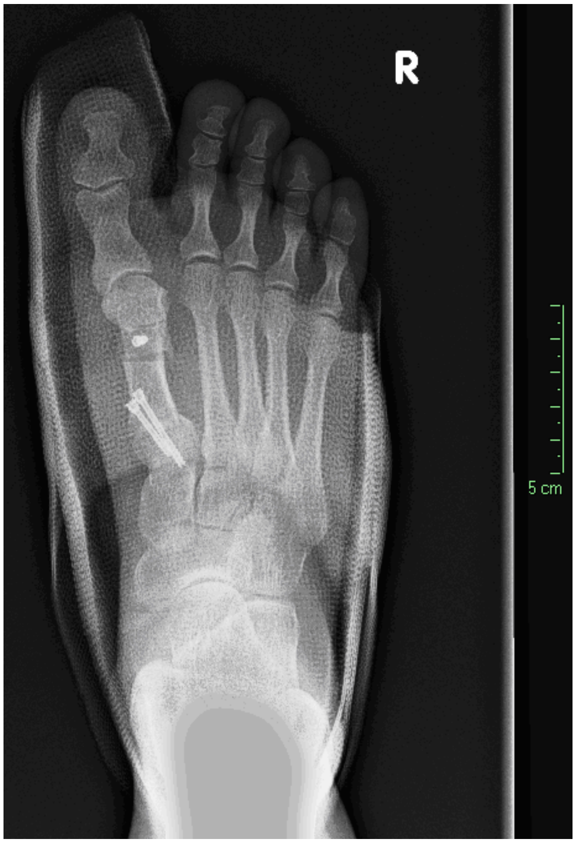

**FIGURE 2.** Anteroposterior postoperative (2 weeks) appearance in Sabot cast.

## DISCUSSION

There are various angular measurements to assess the severity and type of HV deformity, including the HVA, IMA, and DMAA[5,6]. These are combined with an assessment of the MTP joint congruity and degree of osteoarthritis in order to guide surgery on an “à la carte” basis.

The Chevron procedure is a V-shaped translational osteotomy of the first metatarsal head[7,8]. It is suitable for mild HV deformity and patients typically have a HV deformity of <35° and IMA of <15°[8].

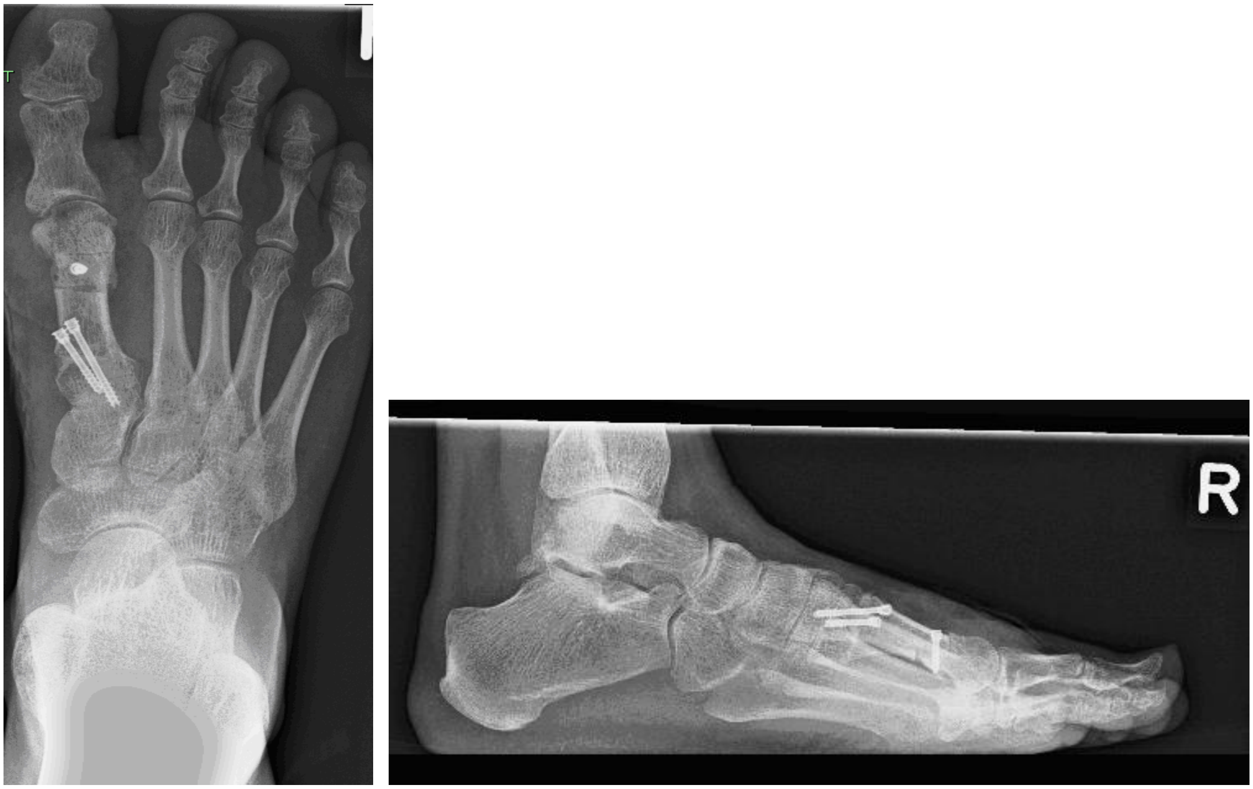

A B

**FIGURE 3.** Three-month postoperative weight bearing of foot; anteroposterior view (A) and lateral view (B).

The Scarf osteotomy takes its name from a carpentry technique to join timber beams longitudinally with maximum strength. It has an advantage of versatility, allowing a wide range of translation and angulation[4]. Although it allows correction of a metatarsus primus varus deformity in primary HV, it was uncertain if a Scarf osteotomy alone would allow correction of both metatarsal primus varus and the DMAA deformity. In appropriately selected patients, Bock et al. suggested that, as a revision procedure, the Scarf osteotomy had a good outcome of clinically and radiographically corrected recurrent HV recurrence in most patients[9].

In this case, the previous Chevron had resulted not only in lateral translation of the metatarsal head, but also increased lateral tilt, represented by an increased DMAA. This awkward deformity required correction by both proximal and distal osteotomies[10,11,12]. Accordingly, two level serial osteotomies were selected: (i) proximal opening-wedge basal osteotomy and (ii) distal short Scarf with medial closing wedges.

## CONCLUSIONS

If a Chevron osteotomy is used inappropriately (for example, in an attempt to correct too large a deformity), it may angulate laterally, causing a malunion with an increased DMAA. It is feasible to correct this combined deformity, of increased DMAA and primus metatarsus varus, by using a combination of proximal opening-wedge and distal Scarf osteotomies. The Scarf osteotomy so used can be short, allowing an angular correction alone at the distal osteotomy of the two serial osteotomies.
